# Supplementary material for: Influence of Nociception Level Monitor (NOL)-Guided Analgesic Delivery on Perioperative Course in Breast Surgeries: A Randomized Controlled Trial
Source: Medicina (Kaunas). 2024 Nov 22;60(12):1921. doi: 10.3390/medicina60121921 (PMC11677679; doi:10.3390/medicina60121921)
Supplement: Supplementary file 1 [file medicina-60-01921-s001.zip › Supplementary file S2. RRDScore chart .pdf]

Recovery room discharge score (RRDS)

Sedation level	Score	Diureses (mL/kg/hour)	Score
Awake	0	>1	0
Part time sleeping, awakening verbal stimuli	1	0.5 - 1.0	1
Part time sleeping, awakening phycical stimuli	2	0 - 0.5	2
Sleeping, no response	3	Anuria	3
**Respiration**		**Blood pressure (mmHg) b**	
Normal (Respiratory frequency ≥10)	0	100 - 160	0
Snoring (Respiratory frequency ≥ 10)	1	90 - 99 or > 160	1
Respiratory frequency < 10	2	81 - 89 or > 170	2
Apnoea periods or obstructive pattern	3	< 80 or > 180	3
**Pain (at rest)**		**Heart rate (beats/minute)**	
None (VAS 0-1)	0	50 – 100	0
Light (VAS 2-4)	1	101 – 120	1
Moderate (VAS 5-7)	2	< 50 or > 120	2
Severe (VAS 8-10)	3	< 40 or > 130	3
**Nausea**		**Temperature (oC)**	
None	0	≥ 36.0	0
Light	1	35.5- 35.9	1
Moderate	2	35.0-35.4	2
Severe	3	<34.9	3
**SpO2(%)a**		**Motor function**	
>94	0	Moves legs freely	0
90-93	1	Moves feet , bend knees	1
85-89	2	Moves only feet	2
<85	3	No movement of legs	3

TableS1. The variables and scores of the RR discharge model. (a) All patients receive 1 to 3 L of nasal oxygen. (b) Blood pressure measured invasively: if using inotropes or vasodilators, the score is increased by 2 points.
